# The Flexion Relaxation Phenomenon in Patients with Radiculopathy and Low Back Pain: A Cross-Sectional Study

**DOI:** 10.3390/jfmk9020077

**Published:** 2024-04-19

**Authors:** Marijan Peharec, Stanislav Peharec, Vedran Srhoj-Egekher, Romana Jerković, Dean Girotto, Gordana Starčević-Klasan

**Affiliations:** 1 Polyclinic of Physical Medicine and Rehabilitation “Peharec”, 52000 Pula, Croatia; peharec.marijan3@gmail.com; 2 Department of Physiotherapy, Faculty of Health Studies, University of Rijeka, 51000 Rijeka, Croatia; stanislav@peharec.com; 3 Faculty of Electrical Engineering and Computing, University of Zagreb, 10000 Zagreb, Croatia; vedran.srhoj-egekher@fer.hr; 4 Department of Anatomy, Faculty of Medicine, University of Rijeka, 51000 Rijeka, Croatia; romana.jerkovic@medri.uniri.hr; 5 Department of Neurosurgery, University Hospital Rijeka, 51000 Rijeka, Croatia; gdeanrow@gmail.com; 6 Department of Basic Medical Science, Faculty of Health Studies, University of Rijeka, 51000 Rijeka, Croatia

**Keywords:** radiculopathy, low back pain, electromyography, kinematic

## Abstract

Although the measurements of the lumbar spine and pelvic flexion have shown that subjects with radiculopathy exhibited greater decreases of motion when compared with subjects with low back pain, there is still a lack of evidence regarding the changes in flexion relaxation ratio in patients with radiculopathy. The aims of this study were to investigate the flexion relaxation ratio and flexion of the lumbar spine and pelvis in subjects with low back pain (LBP) and LBP with radiculopathy (LBPR) in comparison with healthy subjects (CG—control group). A total of 146 participants were divided in three groups: LBP patients (54 males; 21 females); LBPR patients (26 males; 11 females); and CG subjects (16 males; 18 females). The lumbar spine and pelvis flexion was recorded using optoelectronic motion capture system. The electrical activity of the erector spinae muscles was assessed by surface electromyography during flexion-extension movements. Comparisons between groups were made using one-way ANOVA tests and Mann–Whithney U test with the level of statistical significance at 0.05. The lumbar and pelvic flexion and electromyography of the erector spinae muscle showed significant differences between LBP and LBPR patients compared to CG. Patients LBPR showed significantly smaller angles of lumbar and pelvic flexion compared to LBP patients and CG. An increase in the erector spinae muscle activity during flexion was also observed in patients with radiculopathy. The increased muscular activity of the erector spinae is related to the reduced flexion of the lumbar spine in order to protect the lumbar spine structure. Measurements of trunk, lumbar spine and pelvic flexion, and the flexion relaxation ratio may allow us to predict better outcomes or responsiveness to treatment of LBPR patients in the future.

## 1. Introduction

Low back pain (LBP) is a very common condition with lifetime prevalence of 60–80% of the population, with varying degrees of symptoms severity and functional incapacity. Radiculopathy is one of the commonest variations of LBP and is considered responsible for greater work loss, recurrences and costs. Moreover, radiculopathy is considered as a symptom rather than a specific diagnosis defined as dermatomal distribution of pain radiating to the leg with the prevalence of 3% to 5% in the general population [1,2]. Patients with radiculopathy may also have low back pain but this is usually less severe than the leg pain. The diagnosis of radiculopathy is made by correlation of symptoms, clinical signs and imaging findings. Conservative management with lumbar radiculopathy is the most common therapy for most patients and about 90% to 95% will respond to conservative treatment [3,4]. Surgery is indicated in patients with progressive neurological deficits or unmanageable pain [5,6]. Although the recovery prognosis is good, 10% to 30% of patients will develop chronic low back pain and handicap. Suri et al. reported that 25% of patients with radiculopathy experienced a recurrence of the radicular pain symptoms within first year [7].

Kinematic measurement of trunk and pelvis movement, as well as surface electromyography (sEMG), have been suggested as useful methods in the assessment of musculoskeletal dysfunction associated with LBP. These methods have the ability to provide unique information about the functional state of the patient. sEMG measurements can demonstrate changes in activation pattern, fatigability and asymmetrical activation of back muscles in patients with LBP. Healthy people are characterized by significant decrease or silence in erector spinae muscle activity just before and during full forward trunk flexion, which is called flexion-relaxation phenomenon (FRP) [8,9,10]. Silver and Floyd first described absence of the relaxation of erector spinae in chronic LBP patients [11]. The lack of muscle relaxation is the result of pain, neuromuscular adaptation, fear of pain and re-injury in patients with LBP [12,13,14,15]. In addition, subjects with LBP tend to show significantly lower sEMG activity during the extension phase, suggesting that it might reflect neuromuscular abnormalities [16,17]. Previous studies have shown that FRP measurements could be useful for assessment of recovery and efficiency of certain therapeutic procedures in patients with LBP [18,19,20,21,22,23]. Considering the possible changes in muscle activity in different phases of flexion and re-extension and the inability to use sEMG signal normalization in LBP patients compared to healthy subjects, there is a need to calculate the flexion relaxation ratio (FRR) [9,10,11,12,16,17,18,19].

In patients with radiculopathy, the changes in FRR are correlated with disability, positive clinical findings and decreased motion [12,18,20]. Measurements of lumbar spine and pelvic flexion have shown that subjects with radiculopathy exhibited greater decreases of motion when compared with healthy and LBP subjects [21].

In contrast to LBP, there is still lack of evidence regarding the changes in FRR in patients with radiculopathy. Consequently, the objective of current study was to determine whether FRR differences exist between subjects experiencing radiculopathy and those with LBP and healthy subjects. Since the patients with radiculopathy have different movement characteristics, and therefore different responses to interventions in comparison to LBP patients, the aim of this study was to examine which kinematic and sEMG parameters during trunk flexion and extension may differ between patients with radiculopathy and patients with LBP. It was hypothesized that LBPR patients will differ in FRP and EMG muscle activity compared to LBP patients and controls.

## 2. Materials and Methods

### 2.1. Participants

One hundred and twelve LBP and LBPR patients and 34 healthy subjects were screened for participation in the study. The study was carried out in the Polyclinic of Physical Medicine and Rehabilitation, Pula, Croatia. LBP and LBPR patients were recruited from the Polyclinic of Physical Medicine and Rehabilitation, Pula from May 2017 to March 2021. Fifty patients did not fulfill the inclusion criteria. LBP patients (LBP group, n = 75; mean age 39.5 ± 11.1; 54 males; 21 females) were all with pain in the lumbar region lasting more than three months and a negative straight leg raise (SLR) sign. LBPR patients (LBPR group, n = 37; mean age 35.7 ± 12.0; 26 males; 11 females) were all with unilateral or bilateral sciatic dermatome radiating pain from the back into the lower extremity lasting more than three months with or without neurological signs and a positive SLR sign. All LBPR patients had disc herniation at vertebral levels L4–L5 or L5–S1 displaced toward the site of the sciatic nerve diagnosed by magnetic resonance imaging, with normal or slightly degenerative changes and no signs of any spinal pathology that could cause such type of pain. Exclusion criteria were previous spinal deformation, spinal injuries, spinal surgery, spondylolisthesis, osteoporosis, hypermobility, pregnancy and an implanted electrical medical device. Healthy subjects (control group—CG, n = 34; mean age 31.0 ± 10.0; 16 males; 18 females) were recruited from workplaces and community groups by advertising and were eligible for inclusion if they had no history of any LBP episode that required visiting a professional therapy or any other kind of low back disorders. All participants were screened for inclusion and exclusion initially by administrative staff and then re-checked by the assessing clinician. The physical examination and study interventions were delivered by a clinician with 20 years of experience. The sample size calculation was based on the mean value of the root mean square (RMS) for the multifidus muscle in flexion [22]. With an α risk of 0.05, a power of 0.8 and effect size of 0.3, a total of 33 individuals in each group was needed. The groups in our investigation are larger than the required 33 subjects per group because all subjects who requested therapy in the Polyclinic in the period from 2017 to 2021 were included in the research. All participants voluntarily agreed to take part in this research study. The study was conducted in accordance with the Helsinki Declaration of 1975, and was approved by the institutional Ethics Committee of the Polyclinic of Physical Medicine and Rehabilitation Pula (approval #E/02-2017). All participants were adequately informed about the research protocol and provided written consent.

### 2.2. Measures

#### 2.2.1. Subjective Measures Using Questionnaires (LBP and LBPR Groups)

In order to gather information about pain intensity and disability the following standardized questionnaires were used: visual analogue scale (VAS), Oswestry Disability Index (ODI), Roland–Morris Disability Questionnaire (RMDQ), fear avoidance beliefs questionnaire (FABQ). Pain intensity was measured using a 0–10 numerical rating VAS. The anchors used were 0 = “no pain” and 10 = “the worst pain imaginable” [23]. Patients rated the pain intensity they were feeling at the moment of measurement. The RMDQ consists of 24 statements about daily activity limitations due to back pain. Patients were asked to indicate if their LBP interferes with performance of each task at present. Each positive answer is worth one point with scores ranging from 0 = no disability to 24 = severely disabled [24]. The ODI consists of 10 items on the degree of severity to which back (or leg) pain has affected the ability to manage in physical activities including personal hygiene, lifting, walking, sitting, standing, sleeping, sexual activity, social activity and traveling. Each item is rated on a 6-point scale (0–5). The total possible score is 50, with a standardized formula used to transform to a percentage score of disability. The higher score means the higher level of disability related to LBP [23]. FABQ consists of a 4-item FABQ physical activity scale (FABQ-PA, scores potentially ranging from 0 to 24) and a 7-item FABQ work scale (FABQ-W, scores potentially ranging from 0 to 42). Higher values indicate a higher degree of fear-avoidance beliefs for both FABQ scales [25].

#### 2.2.2. Flexion Relaxation Phenomenon

**Figure 1:**
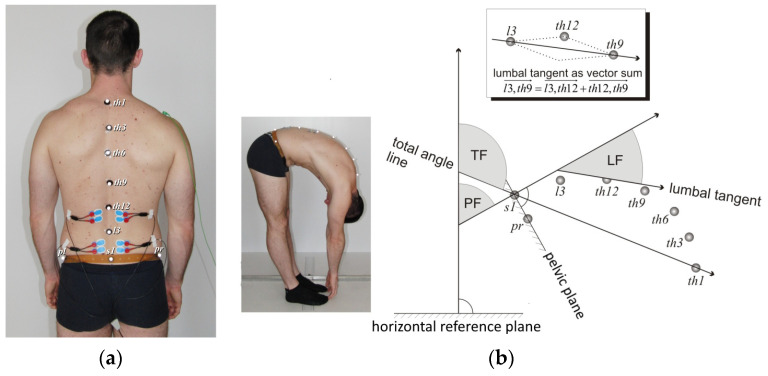
(**a**) Locations of the markers and electrodes for kinematic and sEMG recordings. Two pairs of electrodes applied bilaterally over the lumbar erector spinae muscles at the L1–L2 level and at the L4–L5 level. Six infrared-sensitive markers were placed on spinous processes Th1, Th3, Th6, Th9, Th12, L3, and three were placed on elastic belt and fastened over pelvis landmarks: the anterior superior iliac spine and the posterior superior iliac spine on the left and right sides. (**b**) Position of the participants for trunk flexion analysis. Model showing three variables as an example of the flexed position that was used: total trunk flexion (TF), pelvic flexion (PF) and lumbar spine flexion (LF). Four vectors were used for calculation: vector perpendicular to the horizontal reference plane, vector perpendicular to the pelvis plane, vector starting at S1 and pointing toward T1 for total flexion definition and the lumbar vector between L3–T9.

The protocol for the FRP included kinematic analysis of the low back by means of the motion capture system Smart-D (BTS, Milano, Italy) composed of eight 200 Hz infrared cameras, and recording myoelectric activity of lumbar spine muscles using a wireless surface EMG device, Pocket sEMG biomedical acquisition system (BTS, Milano, Italy). The software version 1.10.0469 provided by the system analyzed synchronously kinematic and EMG of erector spinae muscles signals. For EMG, bipolar disposable surface Ag-AgCl electrodes (Ambu BlueSensor, Ballerup, Denmark) were applied bilaterally over the left and right lumbar erector spinae muscles at the L1–L2 level and at the L4–L5 level approximately 3 cm from the midline, (electrodes were applied perpendicular to the muscle fibers) and with interelectrode spacing of 2.5 cm (Figure 1a) [26]. The skin was previously prepared by gentle abrasion with fine grade paste, and wiping the skin with alcohol swabs. The sEMG activity was recorded with a common mode rejection ratio of >100 dB at 65 Hz, and input impedance of >100 MΩ. Data were collected at a rate of 1000 Hz with a 12-bit A/D converter. The sEMG signals were digitally filtered by a 20–400 Hz band-pass, dual-pass and fourth-order Butterworth filter. The raw sEMG signals were filtered root mean square (RMS) with a 100 ms centered window. Nine infrared sensitive markers of 10 mm in diameter were placed on the subject’s skin to nine anatomical landmarks, superficial to the spinous process of the spine and pelvis [27]. As a part of the marker placement protocol, a belt was placed crossing four anatomical landmarks—the left and right anterior superior iliac spine and also the left and right posterior superior iliac spine. On this belt, two markers—pelvis right (pr) and pelvis left (pl)—were placed symmetrically. Together with a marker placed on the S1 spinous processes, these three points defined a reference plane that represented the pelvis. An additional six markers were placed on the spinous processes L3, Th12, Th9, Th6, Th3 and Th1 (Figure 1a). The participants were asked to perform five trunk flexion-extension tasks (Figure 1b). Verbal instruction, followed by a demonstration and practice trials, were provided before the experiment. From an orthostatic position, participants were instructed to bend forward as far as possible during a 4-s period, they were required to hold the fully flexed position for a 4 s period and return to the initial upright position. The extension phase lasted for 4 s. They stayed in orthostatic position for 4 s. Subjects with radiculopathy were asked to perform flexion until the occurrence of pain in the lumbar region or along the leg.

The data obtained were processed using the Smart Analyzer program (BTS, Milano, Italy) [27,28]. All sEMG signals were measured on the left and right sides of the body. The comparison of individual left-right differences showed no statistically significant differences (paired *t*-test, *p* > 0.050 in all cases); therefore, all sEMG values were replaced by the arithmetic mean of left-right value for each participant. For five cycles of flexion-extension mean peak RMS during flexion, mean RMS in the position of maximum flexion, mean peak RMS during extension, and mean RMS during the orthostatic position of five repetitions, is calculated. From these data, 6 measures of FR were computed. The first two FRR ratios were computed by dividing the normalized mean sEMG activity in maximum trunk flexion (inf) by mean peak sEMG values during flexion movement (f) for two levels of muscles erector spine FRR inf/f L1–L2 and FRR inf/f L4–L5. The second two FRRs computed by dividing the normalized mean sEMG activity in maximum trunk flexion by mean peak sEMG values during extension movement (e) for two levels of lumbar erector spinae muscles FRR inf/e L1–L2 and FRR inf/e L4–L5. The third two FRR computed by dividing the mean peak sEMG activity of lumbar erector spinae muscles during flexion and extension movement, FRR f/e L1–L2 and FRR f/e L4–L5. Details of calculating trunk, lumbar and pelvis angles in sagittal plane are described in Figure 1b.

#### 2.2.3. Measurements

The flexibility of the hamstring muscles was measured in healthy, LBP, and LBPR participants; the SLR sign was measured as well as the hamstring flexibility of the asymptomatic side using a goniometer (Kuntoväline Oy, Helsinki, Finland). The subjects were lying in a supine position and the goniometer was placed on the leg over the lateral last third. The examiner raised the extended leg until the participants felt the hamstrings muscle tension. In participants with radiculopathy and side with positive SLR sign, the examiner raised the affected extended leg until the participants felt pain in the leg.

### 2.3. Statistical Analyses

The statistical data evaluation was performed using STATISTICA version 12.0, StatSoft, Inc. (Los Angeles, CA, USA). Comparisons of continuous variables between three groups (CG, LBP, LBPR, Table 1 and Table 2) were made using one-way ANOVA tests (normality checked by K-S test) and comparisons of ordinal or discrete variables using Mann–Whitney U test (LBP and LBPR, Table 1). The effect size for comparisons in Table 3 was measured by Cohen’s d (determined by calculating the mean difference between two groups, divides by the pooled standard deviation), which was considered large when d > 0.8 [29]. The confounding effects of age, BMI and gender on all measured parameters were checked using multiple regression analyses. The level of statistical significance was set at 0.05 in all analyses.

## 3. Results

**Figure 2:**
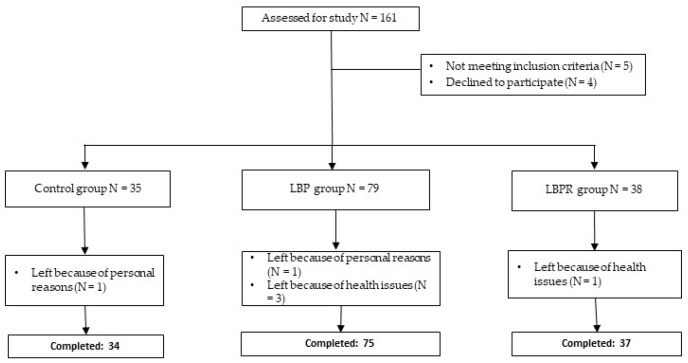
Study CONSORT flow diagram. LBP: low back pain group; LBPR: low back pain with radiculopathy group.

At baseline, 161 subjects were enrolled in the study, and after the exclusion of 15 subjects who did not complete all required assessments, the study was completed with 146 subjects (Figure 2).

The demographic data and clinical status of the examinees are presented in Table 1. The CG participants had a higher proportion of women and were significantly younger (ANOVA, *p* = 0.001, post-hoc Sheffe *p* < 0.001) and had a lower average BMI (ANOVA, *p* = 0.002, post-hoc Sheffe *p* < 0.001) in comparison with LBP and those with LBPR participants, who did not differ significantly in terms of average age or BMI. However, these parameters do not affect the data in Table 1, as the VAS scores and RMQ, ODI, FABQPA and FABQW results are only analyzed between the LBP and LBPR groups, which do not differ by age, BMI and gender. Confounding effects of age, BMI and gender were checked using a regression analysis, which showed no influence on the VAS and questionnaire results presented. Regarding the data in Table 3, no influence of age was found for any of the measured parameters. A slight correlation of BMI was found for the values of total flexion (beta = −1.47, *p* = 0.048, negative correlation as expected), but not for lumbar and pelvic flexion (*p* > 0.05). The same applies to the values of FRR L1–L2 f/e and FRR L4–L5 f/e (beta = 0.015, *p* = 0.045 and beta = 0.016, *p* = 0.047), with higher BMI values leading to slightly higher FRR values, but as mentioned, in the same way for all three groups. As far as the pain status is concerned, the LBPR participants had higher VAS values than the LBP participants did, the difference being significant (median values 6 vs. 3, Mann–Whitney U test, *p* = 0.001 pain for VAS at the measurement and median values 6 vs. 5, *p* = 0.002 for pain VAS average in last 4 weeks). The RMQ score for the LBPR participants was significantly higher than for the LBP participants (median score 12.5 vs. 5, Mann–Whitney U test, *p* < 0.001). The same occurred for the ODI, where the LBPR participants scored significantly higher than the LBP participants (median score 35 vs. 19, Mann–Whitney U test, *p* < 0.001). Furthermore, LBPR participants exhibited higher values in FABQW compared to LBP participants (median score 32.5 vs. 23, Mann–Whitney U test *p* < 0.001) (Table 1). No differences were found also for the degree of pain with respect to the body side. Additionally, a weak but significant correlation was observed between the pain level and FRRs, as shown in Table 2.

The results of the kinematic, FRR, and hamstring flexibility data are presented in Table 3. The hamstrings flexibility significantly differed between all three groups; on the asymptomatic side it was lowest in the LBPR participants, larger in the LBP participants and highest in the CG. All kinematic data for the flexion significantly differed between all three groups. The angle of total flexion was largest in CG participants (116.2), significantly smaller in LBP (99.6) and again significantly smaller in LBPR participants (62.3, ANOVA, *p* < 0.001). The angles of lumbar and pelvic flexion were significantly different in all three groups: smallest in LBPR participants (32.8 and 25.2), larger in LBP (51.9 and 46.5) and largest in CG participants (63.2 and 55.6, ANOVA *p* < 0.001). The Cohen’s effect size calculated between the CG and LBP participants, as well as between CG participants and those LBPR, was large for all of the kinematic data (Table 3). The FRR L1–L2 inf/f, FRR L4–L5 inf/f, FRR L1–L2 inf/e, FRR L4–L5 inf/e, FRR L1–L2 f/e and FRR L4–L5 f/e were significantly different between all three groups. The LBPR participants had the largest FRR values, the LBP participants had smaller values and the CG participants always had the lowest values. Regarding Cohen’s size effect, the LBP participants differed from the CG participants the most in terms of FRR L4–L5 inf/e (0.97), FRR L4–L5 f/e (0.92) and FRR L4–L5 inf/f ratios (0.77). Cohen’s distances of the FRR in the LBPR participants compared to the CG participants were largest for the same ratio, but with larger size effects, which was also true for the comparisons of the LBPR compared to the LBP group (Table 3).

## 4. Discussion

The results of this study have shown that there are significant differences in sEMG measurement of FRR among the three investigated groups. The greatest difference in FRR was noticed between LBPR and CG, whereas the smallest difference was recorded between LBP and CG participants. A comparison of FRR between CG and LBP has shown the most significant difference in FRR L4–L5 inf/e (Cohen’s d 0.97) and FRR L4–L5 f/e (Cohen’s d 0.92). In both comparisons of CG with LBP and CG with LBPR patients the FRR L4–L5 inf/e has shown the most significant difference Cohen’s d 0.97 and 3.53, respectively (Table 3). FRR L1–L2 has shown significantly less difference regarding FRR L4–L5 in all of the three investigated groups. The higher values of lower lumbar erector spinae L4–L5 in comparison to upper lumbar erector spinae L1–L2 might be a result of the immediate vicinity of the source of nociceptive stimulation. These results are consistent with previous studies that have demonstrated that most LBP patients, as well as healthy subjects, have more pronounced FRP changes at the multifidus than at the erector spinae longissimus muscles [30,31]. Although there is no pathophysiological explanation for altered FRP of multifidus muscle, it might be a result of the immediate vicinity of the source of nociceptive stimulation. It is well known that asymmetric atrophy of the multifidus and erector spinae muscles was presented in patients with unilateral LBP or radiculopathy [32]. Due to this fact, asymmetric muscle activation was expected during the measurement of body flexion and extension movements. In this study in all subjects, the muscle activity of the right and left sides was compared. Statistical analysis did not show any significant differences between both sides when analyzing myoelectric characteristics based on RMS calculation. It is worth noticing that such left–right differences can generally be captured by means of sEMG, especially for patients with radiculopathy, as presented in our work [33]. However, this comes at the expense of different sEMG measures being employed (e.g., signal slope change—SSC, permutation entropy—PE, or relative variance difference—RVD) and with more complex feature engineering and analysis involved. As part of this research on the applicability of FRR in differentiating subjects’ groups, the choice of a standard and straightforward measure, such as RMS, was deemed a reasonable entry point. This was supported by detecting statistically significant differences in FRR patterns among groups, calculated on a relevant sample of data.

There are several studies that can explain the change in muscle activity during trunk flexion. Solomonow et al. showed that flexion and stretching of damaged viscoelastic tissue stimulate the pain receptors and cause reflex increased muscle activity of erector spinae in order to protect the injured tissue from load and further damage [34,35]. Intraoperative stretching of compressed and uncompressed spinal nerve root lead to an increased mechanosensitivity of the compressed spinal nerve root [36]. It was noted that inflammation can also cause mechanical sensitivity of the nerve trunk [37]. It has been demonstrated that mechanical provocation during limb movement and the persistent inflammation of neural tissue can cause pain [38,39]. Clinical sign of the presence of an inflammatory process across the sciatic nerve is the SLR test. Hip flexion with a straight leg causes lumbosacral nerve root displacement 0.5 to 5 mm, while trunk flexion causes nerve root displacement up to 3 mm within the intervertebral foramen [40,41,42]. Flexion of lumbar spine and pelvis has demonstrated proximal movement up to 12.2 mm of the tibial branch of the sciatic nerve at the popliteal fossa [43]. Intraoperative measurement showed that nerve root gliding within the intervertebral foramen during SLR testing in patients with intervertebral disc hernia is decreased due to compression while after decompression root gliding is increased [44]. Furthermore, in patients with intervertebral disc herniation, the compression of the nerve root during SLR testing was increased with hip flexion [45]. Increased mechanosensitivity of the nerve due to compression and inflammation of the nerve root and decreased ability of nerve gliding in the intervertebral foramen cause nociceptive stimulation during flexion in patients with radiculopathy. As a response to nociceptive stimulation, with the purpose of protection from possible further tissue injury, reflexive muscle activity of erector spinae occurs and, related to that, limited lumbar and pelvic flexion. Although the muscle activity of hamstrings was not measured, we hypothesized that they also have increased activity, which can limit the flexion of pelvis. It has been proved that painful stimulation of peroneal nerve causes reflex activation of hamstrings [46,47].

Increased muscle activity is associated with active muscle stiffness that is responsible for stiffened and stabilized lumbar spine. The higher muscular activity leads to a greater spinal load [48] and muscle fatigability due to muscles unable to relax normally [49]. Changed muscle activity also might contribute to conversion of low back pain from acute to chronic low back pain.

The results of the present study showed that the trunk, lumbar and pelvis movements are reduced in the LBP and LBPR in comparison with the healthy subjects. A comparison of LBPR and CG participants showed significantly greater difference of the lumbar and pelvis flexion than between LBP and CG participants. Cohen’s d was d −3.10 for lumbar spine and d −3.07 for pelvis in LBPR patients, and d −1.10 for lumbar spine and d −0.85 for pelvis in LBP patients, respectively. Further LBPR participants in comparison with LBP participants exhibited significant decrease in lumbar and pelvic flexion. This limitation may be result of impingement and/or adhesions of the lumbosacral spinal nerve roots limiting the ability of the sciatic nerve to accommodate effectively to spine and pelvis flexion movement. Lumbar spine and pelvic flexion cause greater nociceptive stimulation in LBPR than in LBP participants, which is the reason for increased reflexive muscle activity in order to protect the nerve from stretching. The reduction in pain intensity enables an improvement in movement or functional performance [50,51,52,53]. Our results measuring the FR showed increased muscle activity of the lower lumbar erector spinae due to nociceptive stimulation of the affected tissue, which is responsible for the reduced flexion. The decreased angle of lumbar and pelvic flexion prevents the sciatic nerve from compression and strain during flexion.

Less pain in lumbar region was accompanied by less noticeable kinematic and neurophysiological changes in the LBP participants. Patients with LBP exhibited reduced hamstring flexibility in comparison with healthy subjects. In LBPR patients, the hamstring flexibility of the unaffected side was reduced in comparison with the healthy subjects and LBP participants, and was most probably the result of nervous system excitation. The measurements of the trunk, lumbar and pelvic flexion and FRR and muscle flexibility require the accomplishment of certain tasks during rehabilitation in order to establish physiological mobility, physiological hamstring flexibility, and normal neuromuscular control. Complete movement rehabilitation, movement coordination, muscle flexibility and neuromuscular control will reduce the recurrence, chronicity and absenteeism from work in LBP patients.

This study has several limitations. The first limitation is related to the unequal experimental groups regarding the number and gender of patients included in the experimental protocol. Although the experimental groups are relatively homogeneous in terms of the age of the participants, there are more male than female subjects. In addition, male and female subjects were not specifically analyzed within each experimental group. Moreover, future work should determine if these results correspond to different age groups. Additionally, future work can investigate the applicability of different sEMG measures in detecting intra- and inter-subjects FR relations, specifically tackling left–right differences as well.

The present study suggests that it is important to focus not only on the pain-related factors in LBP and LBPR patients, but also on their abnormal motor control leading to LBP and LBPR disability. Our findings may help to develop clinical interventions that can improve the disability of patients with LBP. Analyzing measurements of FRR, lumbar spine and pelvic flexion may allow us to better predict the outcome or response to treatment of patients with radiculopathy in the future. These results suggest that there are different FRR patterns in healthy individuals, LBP and LBPR patients. This has implications for both further research and treatment and may allow treatments to be targeted to specific patient subgroups. Further research is needed to investigate how such interventions affect FRR, pain intensity and psychological factors in patients with LBP and LBPR.

## 5. Conclusions

This research justified the use of the FRR in evaluating patients with radiculopathy and LBP. The increased muscular activity of the erector spinae is related to the reduced flexion of the lumbar spine. The FRR in patients with radiculopathy and LBP were significantly higher than in healthy subjects, suggesting that this measure may be a useful marker of dysfunctional neuromotor control during the rehabilitation process. Measurement of the trunk, lumbar spine and pelvic flexion, and FRR analysis may allow us in the future to predict better the outcome or responsiveness to treatment of patients with radiculopathy. This may permit the targeting of treatments to particular patient subgroups.

## Figures and Tables

**Table 1:** Demographic data and pain and disability outcomes of the groups.

	CG (n = 34)	LBP (n = 75)	LBPR (n = 37)	*p* (ANOVA)
Age mean ± SD	31.0 ± 10.0	39.5 ± 11.1	35.7 ± 12.0	0.001
BMI mean ± SD	23.0 ± 3.1	25.3 ± 3.4	24.6 ± 2.5	0.002
Gender–female	53%	28%	30%	0.030
				*p* (M-W test)
Pain duration in months *		8.4 (3.6–24.0)	3.6 (1.2–7.2)	0.011
Pain VAS now *		3 (1–5)	6 (2–6)	0.001
Pain VAS average in last 4 weeks *		5 (3–6)	6 (4.5–7)	0.002
RMQ questionnaire *		5 (3–9)	12.5 (8.5–15.5)	<0.001
ODI questionnaire *		19 (10–30)	35 (22–44)	<0.001
FABQPA questionnaire *		18 (14–24)	21 (18–24,5)	0.043
FABQW questionnaire *		23 (9–34.5)	32.5 (25.5–46)	<0.001

Data are mean ± SD, *—median (Q25-Q75), *p*—significance level, CG—healthy subjects, LBP—low back pain subjects, LBPR—LBP subjects with radiculopathy, BMI—body mass index, VAS—visual analogue scale, RMQ—Roland–Morris Disability Questionnaire, ODI—Oswestry Disability Index, FABQPA—fear avoidance beliefs questionnaire-physical activity, FABQW—fear avoidance beliefs questionnaire-work.

**Table 2:** Correlation analysis for pain intensity and muscle activity.

	FRR L1–L2 inf/f	FRR L4–L5 inf/f	FRR L1–L2 inf/e	FRR L4–L5 inf/e	FRR L1–L2 f/e	FRR L4–L5 f/e
VAS now	0.25 (*p* = 0.009)	0.30 (*p* = 0.001)	0.31 (*p* = 0.001)	0.32 (*p* = 0.001)	0.35 (*p* = 0.001)	0.29 (*p* = 0.002)
VAS average in last 4 weeks	0.18	0.17	0.20 (*p* = 0.031)	0.22 (*p* = 0.020)	0.22 (*p* = 0.020)	0.26 (*p* = 0.002)

*p*—significance level, VAS—visual analogue scale, L1–L2—erector spine muscle at level L1 and L2, L4–L5 erector spine muscle at level L4 and L5. f—mean peak RMS during flexion, inf—mean RMS in the position of maximum flexion, and e—mean peak RMS during extension.

**Table 3:** Kinematic, electromyographic and flexibility data.

	I CG (n = 34)		II LBP (n = 75)		III LBPR (n = 37)		*p*	Cohen’s *d*		
MOTION	Mean	SD	Mean	SD	Mean	SD		LBP to CG	LBPR to CG	LBP to LBPR
Total flexion /°	116.2	9.9	99.6	16.4	62.3	19.7	<0.001	−1.14	−3.44	−2.17
Lumbar flexion /°	63.2	6.5	51.9	11.7	32.8	12.2	<0.001	−1.10	−3.10	−1.66
Pelvis flexion /°	55.6	7.0	46.5	12.1	25.2	12.1	<0.001	−0.85	−3.07	−1.80
Hamstrings flexibility right /°	86.3	9.6	77.8	10.0	71.0	13.6	<0.001	−0.90	−1.84	−1.44
Hamstrings flexibility left /°	86.6	9.6	77.3	9.8	70.7	13.5	<0.001	−0.97	−2.08	−1.67
**sEMG**	**Mean**	**SD**	**Mean**	**SD**	**Mean**	**SD**	***p***	**LBP to CG**	**LBPR to CG**	**LBP to LBPR**
FRR L1–L2 inf/f	0.25	0.13	0.35	0.27	0.75	0.30	<0.001	0.43	2.18	1.30
FRR L4–L5 inf/f	0.25	0.13	0.46	0.32	0.85	0.22	<0.001	0.77	3.27	1.36
FRR L1–L2 inf/e	0.07	0.03	0.16	0.16	0.46	0.26	<0.001	0.66	2.09	1.56
FRR L4–L5 inf/e	0.09	0.05	0.29	0.25	0.68	0.23	<0.001	0.97	3.53	1.74
FRR L1–L2 f/e	0.32	0.14	0.41	0.15	0.59	0.21	<0.001	0.61	1.53	1.14
FRR L4–L5 f/e	0.40	0.11	0.55	0.18	0.79	0.15	<0.001	0.92	2.89	1.33

CG—healthy subjects, LBP—low back pain subjects, LBPR—LBP subjects with radiculopathy, SD—standard deviation, *p*-significance level *p* ≤ 0.05, L1–L2—erector spine muscle at level L1 and L2, L4–L5 erector spine muscle at level L4 and L5. f—mean peak RMS during flexion, inf—mean RMS in the position of maximum flexion, and e—mean peak RMS during extension.

## Data Availability

Data supporting this article are available from the corresponding author on reasonable request.
